# Robotic-assisted colorectal surgery increases the adherence to enhanced recovery concepts

**DOI:** 10.1007/s00464-026-12828-z

**Published:** 2026-05-08

**Authors:** Johanna C. Wagner, Lena Wagner, Anna Widder, Regina Pistorius, Matthias Kelm, Nicolas Schlegel, Florian Seyfried, Armin Wiegering, Christian Markus, Patrick Meybohm, Christoph-Thomas Germer, Wolfgang Schwenk, Sven Flemming

**Affiliations:** 1 Department of General, Visceral, Vascular and Pediatric Surgery, University Hospital Würzburg, Oberdürrbacherstr. 6, 97080 Würzburg, Germany; 2 Department of Anaesthesiology, Intensive Care, Emergency and Pain Medicine, University Hospital Würzburg, Würzburg, Germany; 3 GOPOM GmbH, Gesellschaft Für Optimiertes PeriOperatives Management, Düsseldorf, Germany; 4 Department of General, Visceral and Thoracic Surgery, University Hospital Frankfurt, Frankfurt, Germany

**Keywords:** Enhanced recovery concept, Robotic-assisted surgery, Colorectal surgery

## Abstract

**Background:**

The implementation of enhanced recovery concepts (ERC) has been shown to improve postoperative patients’ outcomes. One key aspect of ERCs is the minimally invasive approach to the surgical field. To date, data showing potential advantages of robotic-assisted over laparoscopic surgery within ERCs are scarce.

**Methods:**

All patients receiving minimally invasive colorectal surgery between January 2021 and February 2023 at the University Hospital Würzburg, Germany were prospectively included and divided into two groups according to the type of surgery being performed (laparoscopic versus robotic-assisted). The primary outcome was adherence to the ERC protocol. Secondary endpoints were postoperative complications, length of hospital stay and the rate of readmission within a follow-up of 30 days postoperatively.

**Results:**

164 patients were included, of which 48.8% (*n* = 80) received laparoscopic and 51.2% (*n* = 84) robotic-assisted surgery. Operating times were longer in the robotic-assisted cohort, but the conversion rate as well as the postoperative complication rate and blood loss were comparable in both groups. The overall adherence to the ERC protocol was higher in the robotic-assisted cohort compared to the laparoscopic surgery cohort (80.4% vs. 65.2%, *p* = 0.005). Patients’ full autonomy was reached earlier in patients after robotic-assisted surgery (4 vs. 5 days, p = 0.048). Furthermore, systemic inflammation parameters were lower in patients after robotic-assisted surgery.

**Conclusion:**

Robotic-assisted surgery may improve adherence to ERCs and could reduce postoperative systemic inflammation, potentially contributing to faster postoperative recovery compared with the laparoscopic approach.

For the past three decades, enhanced recovery after surgery (ERAS®) or “Fast Track” concepts have been increasingly established in the surgical field. Enhanced recovery concepts (ERC) are multimodal, evidence-based protocols which have been shown to improve postoperative outcomes [1]. ERCs contain protocols for the preoperative, intraoperative, and postoperative periods. The key concepts are the preoperative optimization of the patient’s health, the minimization of the perioperative stress and the fast postoperative recovery. Thus, preoperative recommendations include reducing patients’ comorbidities by cessation of alcohol consumption and smoking, improving preexisting anemia, increasing fitness levels and optimizing nutritional intake. Intraoperatively, recommendations focus on maintaining normothermia, normovolemia and homeostasis, and the usage of minimally invasive surgical approaches [1]. Early mobilization, avoidance or early removal of drains and catheters and the optimization of pain control are central recommendations for the postoperative period. The implementation of ERCs in different fields of surgery has been proven to reduce postoperative morbidity and mortality [2–7].

One key aspect of ERCs is the minimally invasive access to the surgical field. In 1980, the first laparoscopic appendectomy was performed and has since revolutionized the surgical field [8]. Nowadays, the laparoscopic approach has become the standard of care and has replaced the open approach also for complex surgeries. Laparoscopic surgery has been shown to reduce postoperative pain, improve morbidity and mortality, and decrease the length of postoperative hospital stay [9–13]. Studies have also shown an improvement in short-term outcomes with comparable oncological outcomes between the laparoscopic and open surgical techniques [14–16]. These advantages of laparoscopic surgery have led to the ERC-recommendation of using minimally invasive surgeries, when feasible [17].

In recent years, further technological advances have introduced robotic-assisted surgery. Robotic-assisted surgery is a minimally invasive surgical technique, which brings additional advantages, such as better visualization, articulating instruments, better ergonomics, and enhanced dexterity. Studies have shown a shorter length of postoperative stay and a decreased conversion rate when comparing the robotic technique to the laparoscopic technique [18, 19]. The proportion of robotic surgery has been steadily increasing since 2013, which has led to a decrease in open and laparoscopic surgery [18].

Despite increasing numbers of robotic-assisted surgeries, potential advantages in comparison to the laparoscopic approach within established enhanced recovery concepts have not been extensively studied. To elucidate potential differences between these surgical approaches, we analyzed the perioperative outcomes after robotic-assisted and laparoscopic colon resections in an established enhanced recovery concept.

## Methods

All patients receiving minimally invasive colorectal surgery between January 2021 and February 2023 at the Department of Visceral Surgery of the University Hospital Würzburg, Germany were prospectively included in this cohort study. The ERC program was implemented in 2021 and all patients were included in the program. The patients were divided into two groups according to the type of minimally invasive surgery (laparoscopic versus robotic-assisted). Patients were not specifically selected for laparoscopic or robotic-assisted surgery. The selection was based on operating room capacity. All surgeries were performed by three specialized colorectal surgeons, equally divided between the laparoscopic and robotic-assisted surgical approaches.

Sociodemographic and clinicopathological data including diagnosis, medication and comorbidities were prospectively collected. Surgical data, including operating time, intraoperative blood loss, the rate of conversion as well as postoperative complications, measured by the Comprehensive Complication Index (CCI), were also analyzed. The adherence to the enhanced recovery concept was analyzed. This concept included the pre-, intra-, and postoperative phases and the detailed list of items of the enhanced recovery concept is summarized in Table 1. Trained ERC nurses documented the adherence to each of these items for each patient in a prospective manner.

**Table 1:** Items and goals of the enhanced recovery concept for the pre-, intra-, and postoperative periods

Preoperative items	Patient information by specialized ERC assistants
	Prehabilitation and optimization of patient’s health status, including cessation of smoking or alcohol consumption 4 weeks prior to surgery
	Nutritional risk assessment and therapy of malnutrition including carbohydrate drinks
	Anemia screening and management: Blood count, if low, then substitution of iron and/or vitamin B12
	Hospital admission on day of surgery
	Prevention of nausea and vomiting
	No anxiolytic premedication
	Euvolemia
	No prolonged preoperative fasting and carbohydrate loading (e.g., Pre-OP Nutricia: 800 ml the day prior surgery and 400 ml 2 h before the surgery)
	Antibiotic decontamination (p.o.) prior to surgery
Intraoperative items	Prophylactic i.v. antibiotics before skin incision
	Standardized anesthesia protocol
	Prophylaxis of postoperative nausea and vomiting (PONV)
	Euvolemia and normothermia
	Regional anesthesia if needed
	Adequate analgesia without long-lasting opioids
	No gastric tube placement or removal at the end of the surgery
	Avoidance of drainage placement
	Fluid management the day of surgery: max. 3000 – 3500 ml, ideally no postoperative i.v. fluids
Postoperative items	No gastric tube
	Multimodal analgesia
	Thromboprophylaxis
	Euvolemia and no i.v. fluids
	Rapid removal of urinary catheter, if still in place
	Prophylaxis of gastrointestinal atony
	Immediate oral nutrition, including high protein drinks (day of surgery: 200 kcal, postoperative day 1: 400 kcal, days 2 + 3: 600 kcal)
	Rapid removal of the peridural catheter (latest postoperative day 3)
	Early mobilization (Operation day: > 15 min; day 1: > 4 h, day 2: > 6 h, day 3: > 8 h)
	Follow-up 30 days postoperatively by specialized ERC assistants

The primary outcome was adherence to the enhanced recovery concept. The adherence was calculated according to the goals met, which were defined by the ERC program (Table 1). The documentation of the adherence to the individual enhanced recovery elements was only done for certain items, thus, leaving this dataset incomplete. Secondary endpoints were postoperative complications, the length of hospital stay, the rate of readmission within a follow-up of 30 days postoperatively and the MTL30 (mortality, transfer, length of hospital stay within 30 postoperative days) [20, 21]. In addition, the postoperative serum levels of leukocytes and C-reactive protein (CRP) were analyzed.

Statistical analysis was performed using IBM SPSS Statistics for Windows, version 28.0 (IBM Corp., Armonk, NY, USA). Descriptive data are presented as mean with standard deviation, median with range, or total numbers with percentage. Differences in patient characteristics were assessed by the Chi-squared test, Fisher’s exact test, or ANOVA test according to the data scale and distribution. Multivariate analyses of variance were performed by using MANOVA (dependent variables: laparoscopic surgery, robotic-assisted surgery). The tested variables in the multivariate analysis were the duration of surgery, the readiness for discharge, the CRP- and leukocyte values on postoperative days 3–4, the CCI, and the overall adherence > 70%. Statistical relevance was considered at a *p*-value < 0.05.

Ethical approval for the study was obtained from the Ethics Committee of the University of Würzburg, Germany. The study was registered in the Research Registry (researchregistry10599).

## Results

### Patient characteristics

In this prospective single-center study, 164 consecutive patients received colorectal surgery between January 2021 and February 2023 and were enrolled in the ERC program. Within this patient cohort, 48.8% (*n* = 80) underwent laparoscopic surgery and 51.2% (*n* = 84) robotic-assisted surgery. More female patients were included in the robotic-assisted surgery group (robotic: 59.5%; laparoscopic: 38%; *p* = 0.006). All other baseline characteristics between the two patient cohorts did not differ (Table 2).

**Table 2:** Patient characteristics

	Laparoscopic surgery *n* = 80 (48.8%)	Robotic-assisted surgery *n* = 84 (51.2%)	*p*-value
Age [years], median (range)	64.5 (28–88)	61 (25–89)	0.299
Sex (F:M), *n* (%)	30 (38): 49 (62)	50 (59.5): 34 (40.5)	**0.006**
BMI [kg/m^2^], median (range)	26.2 (16.4–48.3)	25.9 (16.4–37.7)	0.299
ASA classification, median (range)	2 (1–3)	2 (1–4)	0.129
CCI, mean (SD)	4.0 (2.7)	3.8 (2.9)	0.663
Cardiovascular risk factors, *n* (%)			0.673
Coronary heart disease /STEMI/NSTEMI	8 (10.1)	8 (9.6)	
Heart failure	4 (5)	1 (1.2)	
Other	32 (40)	34 (40.5)	
Liver disease, *n* (%)			0.116
Fibrosis/MASLD	2 (2.5)	0	
Cirrhosis	2 (2.5)	0	
Chronic kidney disease, *n* (%)	6 (7.5)	3 (3.6)	0.269
Immunosuppressive medication, n (%)	4 (5)	4 (4.8)	0.944

Bold values denote significant *p*-values

*ASA* American Association of Anesthesiologists, *BMI* Body mass index, *CCI* Comprehensive comorbidity Index, *MASLD* Metabolic dysfunction associated steatotic liver disease, *NSTEMI* non-ST-elevated myocardial infarction, *STEMI* ST-elevated myocardial infarction

### Surgical parameters

The most common indication for surgery was a colorectal carcinoma (*n* = 87, 53%), followed by symptomatic diverticular disease (*n* = 67, 40.9%) (Table 3). The surgical procedures performed varied from right- or left-sided hemicolectomy to sigmoid and rectal resections (see Table 3). The conversion rate to open surgery did not differ between the patient cohorts (laparoscopic 13.8%; robotic 8.3%, *p* = 0.267) and the mean intraoperative blood loss was comparable (robotic: 125.8 ml; laparoscopic: 155.3 ml; *p* = 0.206). Similarly, the percentage of ostomy placement did not differ between the two groups (robotic: 23.8%; laparoscopic: 31.1%; *p* = 0.299). The surgical time, however, was longer in the robotic-assisted group compared to the laparoscopic surgery group (median 237.5 vs. 181.5 min, *p* = 0.001) (Table 3).

**Table 3:** Surgical parameters

	Laparoscopic surgery *n* = 80 (48.8%)	Robotic-assisted surgery *n* = 84 (51.2%)	*p*-value
Indication for surgery, *n* (%)			0.677
Colorectal carcinoma	45 (56.3)	42 (50)	
Diverticulitis	31 (38.8)	36 (42.9)	
Other	4 (5)	6 (7.1)	
Surgical procedure, *n* (%)			0.952
Ileocecal resection	1 (1.3)	0	
Right-sided hemicolectomy	8 (10)	8 (9.5)	
Left-sided hemicolectomy	19 (23.8)	16 (19)	
Sigmoid resection	26 (32.5)	31 (36.9)	
Anterior rectal resection	7 (8.8)	10 (11.9)	
Low anterior rectal resection	14 (17.5)	14 (16.7)	
Abdominoperineal rectal excision	3 (3.8)	3 (3.6)	
Other	2 (2.5)	2 (2.4)	
Ostomy placement, *n* (%)	25 (31.3)	20 (23.8)	0.299
Conversion rate, *n* (%)	11 (13.8)	7 (8.3)	0.267
Blood loss {mL], mean (SD)	155.3 (178.3)	125.8 (108.0)	0.206
Operating time [min], median (range)	181.5 (75–364)	237.5 (70–480)	**0.001**

Bold values denote significant *p*-values

### Adherence to the enhanced recovery concept

All patients were included in an enhanced recovery concept program. The overall adherence was significantly higher in the robotic-assisted cohort compared to the laparoscopic surgery cohort (80.4% vs. 65.2%, *p* = 0.005; Table 4). When analyzing the pre-, intra-, and postoperative adherence, the main difference between the two patient cohorts resulted from the intra- and postoperative adherence (intra: 85.7% vs. 71.4%, *p* = 0.001; post: 64.3% vs. 42.9%, *p* = 0.020; Table 4). Although we do not have recorded data of the adherence to all individual enhanced recovery elements, we do show that certain elements show higher adherence in the robotic-assisted approach, such as the intraoperative drain placement and postoperative i.v. fluid therapy, early removal of the urinary catheter, or postoperative ileus prophylaxis (Table 5). In addition, patients after robotic-assisted surgery were fully autonomous, and thus ready for discharge, significantly earlier than patients after laparoscopic surgery (4 vs. 5 days, *p* = 0.048; Table 4).

**Table 4:** Adherence to the enhanced recovery concept

	Laparoscopic surgery *n* = 80 (48.8%)	Robotic-assisted surgery *n* = 84 (51.2%)	*p*-value
Adherence [%], median (range)			
Preoperative	77.8 (22–100)	88.9 (33–100)	0.137
Intraoperative	71.4 (43–100)	85.7 (43–100)	**0.001**
Postoperative	42.9 (0–100)	64.3 (0–100)	**0.020**
Overall	65.2 (30–96)	80.4 (43–100)	**0.005**
First bowel movement [days], median (range)	2 (1–7)	1 (0–5)	0.050
Intake of solid foods [days], median (range)	3 (0–14)	2 (0–12)	0.067
Mobilization [h], median (range)			
Postop day 1	5 (0–9)	5 (1–12)	0.422
Postop day 2	7 (2–12)	7.5 (1–14)	0.946
Postop day 3	9 (0–13)	9.5 (0–16)	0.701
Length of hospital stay [days], median (range)	7.5 (2–45)	5 (2–48)	0.091
Readiness for discharge [days], median (range)	5 (0–31)	4 (0–42)	**0.048**

Bold values denote significant *p*-values

**Table 5:** Enhanced recovery elements for the intra- and postoperative adherence

Enhanced recovery elements	Laparoscopic surgery	Robotic surgery	p-value
	% adherent patients	% adherent patients	
Intraoperative			
Short acting opioids	32	22	0,21
Adequate regional anesthesia	70	95	** < 0.001**
Nasogastric tube ex at end of surgery	97	100	0.21
No drain	67	90	**0.007**
Postoperative			
Adequate IV fluid therapy	55	72	**0.03**
Early oral nutrition	36	51	0.08
Prophylaxis of postoperative ileus	69	86	**0.012**
Early mobilization	47	60	0.11
Early removal of urinary catheter	65	88	**0.001**

Bold values denote significant *p*-values

### Postoperative outcome

**Fig. 1:**
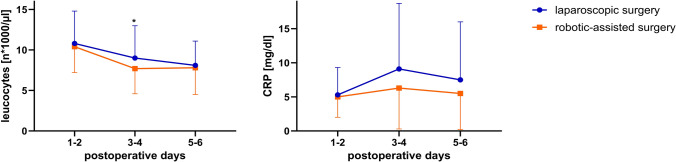
Postoperative leukocyte count and CRP-values measurements of leukocyte counts and CRP-values on postoperative day 1–2, 3–4 and 5–6 show significantly lower values for patients after robotic-assisted surgery on postoperative day 3–4 (leukocytes: lap 9.0 ± 4.0, rob 7.7 ± 3.1, *p* = 0.025; CRP: lap 9.1 ± 9.6, rob 6.3 ± 6.0, *p* = 0.031). *CRP*, C-reactive protein

Postoperative complications and the Comprehensive Complication Index (CCI) did not differ between the two patient cohorts (Table 6). The MTL30 (*n* = 5, 3.0%) was comparable in both patient cohorts (Table 6). Interestingly, leukocyte values and C-reactive protein (CRP) on postoperative days 3–4 were significantly lower after robotic-assisted surgery (leukocytes: 7.7 vs. 9.0 *1000/µl, *p* = 0.025; CRP: 6.3 vs. 9.1 mg/dl, *p* = 0.031) (Fig. 1). Multivariate analysis confirmed that robotic-assisted surgery is accompanied by a longer operation time, decreased CRP-values on postoperative days 3–4 and improved adherence to the enhanced recovery concept (Table 7).

**Table 6:** Postoperative complications

	Laparoscopic surgery *n* = 80 (48.8%)	Robotic-assisted surgery *n* = 84 (51.2%)	*p*-value
Postoperative complications, *n* (%)			
Pneumonia	1 (1.3)	1 (1.3)	0.972
Thrombosis/pulmonary embolism	0	1 (1.2)	0.328
Anastomotic leakage	5 (6.3)	4 (4.8)	0.676
Wound infection	7 (8.8)	8 (9.5)	0.864
Bleeding	3 (3.8)	3 (3.6)	0.951
Blood transfusion	7 (8.8)	3 (3.6)	0.166
Re-operation	9 (11.3)	6 (7.1)	0.362
CCI, mean (SD)	11.5 (16.9)	7.2 (13.9)	0.077
Clavien-Dindo > 2, *n* (%)	14 (17.5)	9 (10.7)	0.211
MTL30, *n* (%)	2 (2.5)	3 (3.6)	0.690

*CCI* Comprehensive complication Index, *MTL30* mortality, transfer, length of stay > 30 days

**Table 7:** Multivariate analysis comparing the laparoscopic to the robotic-assisted group

	Odds ratio	95% CI	*p*-value
Operation time	1.02	1.012 – 1.027	**0.001**
CRP, postoperative day 3–4	0.925	0.857 – 0.999	**0.047**
Leucocytes, postoperative day 3–4	0.974	0.842 – 1.128	0.728
CCI > 26.2	0.785	0.078 – 7.931	0.838
Readiness for discharge	0.942	0.865 – 1.026	0.171
Adherence	3.383	1.531 – 7.475	**0.003**

Bold values denote significant *p*-values

## Discussion

The successful implementation of enhanced recovery concepts heavily relies on the adherence to the pre-, intra-, and postoperative protocols. A survey among healthcare providers in 2016 emphasizes the difficulties in the implementation of an enhanced recovery concept. The main barriers for a successful implementation were time restraints (69%), opposing colleagues (68%), and logistical problems (66%) [22]. In the available literature, the adherence to enhanced recovery concept protocols varies from 35 to 94% [4, 18, 23, 24].

Our study showed an improved overall adherence to the established enhanced recovery concept protocol in patients with robotic-assisted compared to laparoscopic surgery. In our data set, the difference of the intraoperative adherence is not due to the surgical approach (i.e., drain placement) but rather intraoperative findings. However, certain postoperative elements, such as sufficient oral fluid intake, prophylaxis of postoperative ileus (i.e., chewing gum, mobilization, coffee intake), and the early removal of the urinary catheter were achieved significantly more frequently in the robotic-assisted approach. This is in contrast to one large study comparing open, laparoscopic and robotic-assisted rectum resections showing an increased adherence to enhanced recovery concepts for the minimally invasive but not a further increase for the robotic-assisted approach [18]. Nevertheless, when postoperative adherence was analyzed in more detail—particularly regarding postoperative pain management—the robotic-assisted approach showed advantages over the laparoscopic approach [18]. These findings suggest that while overall protocol adherence may not consistently differ between minimally invasive techniques, specific postoperative components of enhanced recovery pathways might benefit from the robotic-assisted approach. One good example is the more frequent early removal of the urinary catheter shown in the robotic-assisted group. Literature has shown that the postoperative bladder and sexual function are preserved significantly more often after robotic-assisted surgery, potentially explaining this difference between the two surgical approaches [25–27].

Other criteria of postoperative adherence are the readiness for discharge and the length of hospital stay. We show a trend toward a decrease in duration of hospital stay and an earlier readiness for discharge in the robotic-assisted surgery group. However, the interpretation of discharge timing must consider the German hospital reimbursement system, which currently requires a minimum postoperative hospital stay of five days after colorectal surgery. Consequently, patients were not necessarily discharged at the time they were deemed clinically ready for discharge. Despite this structural limitation, the robotic-assisted group demonstrated a notably shorter length of hospital stay (5 days) compared with both the laparoscopic group in our study (7.5 days) and previously reported national and international benchmarks. Data from the German StuDoQ registry report hospital stays of 7–8 days between 2017 and 2021, while international cohorts report an average of approximately 6.5 days [28]. This reduction was not observed in the laparoscopic group, which had a median hospital stay of 7.5 days. Similarly, a Swedish cohort of patients after rectum resection, showed the similar results with less symptoms delaying discharge and a shorter length of hospital stay when comparing the robotic-assisted to the laparoscopic surgical approach [18]. The importance of enhanced recovery concepts in the context of minimally invasive surgery was also shown in the LAFA trial [29]. This multicenter, randomized controlled study demonstrated that laparoscopic surgery combined with fast-track care led to the shortest hospital stays and fastest recovery, whereas neither intervention alone achieved the same magnitude of benefit [29]. Thus, integrating minimally invasive techniques with structured enhanced recovery pathways optimizes postoperative recovery. Taken together, these results reinforce the concept that higher compliance with enhanced recovery protocols is associated with shorter hospital stay and reduced severity of postoperative complications [4, 30, 31].

In recent years, robotic-assisted surgery has been increasingly applied, despite limited evidence of substantial advantages over the laparoscopic approach [32]. Many studies thus far have shown significantly longer operating times with the robotic-assisted approach [33, 34], a finding that was also observed in our cohort. Despite longer operating times, we did not observe an increase in postoperative complications in the robotic-assisted group and, importantly, found a significantly earlier readiness for discharge, suggesting a potential benefit in postoperative recovery. Although the ROLARR trial in 2018 reported no difference between the laparoscopic and robotic-assisted approaches for rectal resections [35], other studies have shown a decreased rate of conversion to open surgery and a significant improvement of patient outcome after robotic-assisted surgery [33, 34]. Moreover, the REAL-trial demonstrates that robotic-assisted surgery for resection of the middle and lower rectum results in better oncological quality of resection, an amelioration of postoperative recovery, and a decreased 3-year locoregional recurrence rate compared to the laparoscopic approach [25]. Taken together, these findings suggest that although robotic-assisted surgery may require longer operative times, it may offer clinically relevant benefits that become particularly evident in postoperative recovery and oncological outcomes.

Despite these advantages of robotic-assisted surgery, the reason for the improved outcome after robotic-assisted surgery over laparoscopic surgery remains unclear. One potential explanation could be the previously reported lower levels of systemic inflammation, indicated by lower postoperative inflammation markers (leukocyte counts and CRP-values) [19, 36, 37]. In line with these results, we demonstrate a significantly reduced postoperative stress response, characterized by lower levels of leukocyte counts and CRP-values in the patient cohort after robotic-assisted surgery. Reduced surgical trauma and systemic inflammation might therefore contribute to faster postoperative recovery and facilitate improved adherence to enhanced recovery protocols, which might ultimately translate into shorter hospital stays and improved patient outcomes.

Our work has several limitations, including the lack of randomization and blinding, which may introduce selection bias and limit the generalizability of the results. Additionally, the number of patients is small, and all patients were treated at a single tertiary referral center, which may reflect center-specific practices and expertise. These factors could influence both surgical outcomes and adherence to enhanced recovery protocols, and therefore the results should be interpreted with caution. Despite these limitations, our study is among the first prospective analyses to systematically evaluate perioperative enhanced recovery concepts in patients undergoing both laparoscopic and robotic-assisted colorectal resections. Importantly, by directly comparing these two minimally invasive approaches within a structured enhanced recovery framework, our findings provide novel insights into the potential benefits of robotic-assisted surgery, including improved protocol adherence, attenuated postoperative inflammatory response, and earlier readiness for discharge. These results contribute evidence to the ongoing discussion on optimizing perioperative care in colorectal surgery and suggest that robotic-assisted techniques might play a complementary role in enhancing recovery programs. Future multicenter, randomized studies with larger cohorts are warranted to validate our observations and to further clarify the mechanisms by which robotic-assisted surgery may facilitate postoperative recovery.

## Conclusion

In summary, the use of the robotic-assisted surgical approach may improve the adherence to enhanced recovery concepts and the patient’s readiness for discharge. In addition, the robotic-assisted surgical approach may reduce postoperative systemic inflammation, and, thus, robotic-assisted surgery might have advantages over the laparoscopic approach in postoperative patient outcomes.
